# The reporting of pilot and feasibility studies in the top dental specialty journals is suboptimal

**DOI:** 10.1186/s40814-022-01182-1

**Published:** 2022-10-04

**Authors:** Mohammed I. U. Khan, Hartirath K. Brar, Cynthia Y. Sun, Rebecca He, Hussein A. El-Khechen, Katie Mellor, Lehana Thabane, Carlos Quiñonez

**Affiliations:** 1 Department of Dental Public Health, University of Toronto, Toronto, Ontario Canada; 2 Biostatistics Unit, St. Joseph’s Healthcare, Hamilton, Ontario Canada; 3 Department of Materials and Biomedical Engineering, McMaster University, Hamilton, ON Canada; 4 Department of Health Sciences, McMaster University, Hamilton, ON Canada; 5 Department of Health Research Methods, Evidence, and Impact, McMaster University, Hamilton, ON Canada; 6 Centre for Statistics in Medicine, Nuffield Department of Orthopaedics, Rheumatology and Musculoskeletal Sciences, University of Oxford, Oxford, UK

**Keywords:** Pilot studies, Feasibility studies, Dentistry, Dental specialties

## Abstract

**Background:**

Pilot and feasibility studies (PAFS) are smaller investigations seeking to assess the feasibility of conducting a larger more definitive study. In late 2016, the CONSORT statement was extended to disseminate good practices for reporting of randomized pilot and feasibility trials. In this quality assurance review, we assessed whether PAFS in the top dental speciality journals adhere to good practices of conduct and reporting, by prioritizing assessment of feasibility and stating pre-defined progression criteria to inform the decision to pursue funding for a larger trial.

**Methods:**

With the help of a librarian, we searched MEDLINE and EMBASE from 2017 to 2020, inclusive, for PAFS in the top 3 journals from each of the 10 dental specialties. We collected data on methodological and general characteristics of the studies, their objectives, and reporting of items recommended in the CONSORT extension.

**Results:**

Of the 111 trials included, 51.4% (95% CI 41.7–61.0%) stated some indication of intent to assess feasibility while zero reported progression criteria; 74.8% (95% CI 65.6–82.5%) of trials used the terms “pilot” or “feasibility” in their titles and 82.9% (95% CI 74.6–89.4%) of studies stated there is a need for a future trial, but only 9.0% (95% CI 4.4–15.9%) stated intent to proceed to one. Most of the studies, 53.2% (95% CI 43.4–62.7%), reported hypothesis testing without cautioning readers on the generalizability of the results. Studies that used the terms “pilot” or “feasibility” in their title were less likely to have feasibility objectives, compared to trials that did not, with an odds ratio (OR) of 0.310 (95% CI 0.103–0.930; *p* = 0.037). Compared to trials that did not conduct hypothesis testing, trials that conducted hypothesis testing were significantly less likely to assess feasibility, among them, trials that cautioned readers on the generalizability of their results had an OR of 0.038 (95% CI 0.005–0.264; *p* < 0.001) and trials that did not caution readers on the generalizability of their results had an OR of 0.043 (95% CI 0.008–0.238; *p* = 0.001).

**Conclusion:**

Many PAFS in dentistry are not conducted with the intent of assessing feasibility, nor do they state progression criteria, and few report intent to proceed to a future trial. Misconceptions about PAFS can lead to them being poorly conducted and reported, which has economic and ethical implications. Research ethics boards, funding agencies, and journals need to raise their standards for the conduct and reporting of PAFS, and resources should be developed to address misconceptions and help guide researchers on the best practices for their conduct and reporting.

**Supplementary Information:**

The online version contains supplementary material available at 10.1186/s40814-022-01182-1.

## Key messages regarding feasibility

- The quality of conduct and reporting of pilot and feasibility studies in the field of dentistry is unclear.
- The conduct and reporting of pilot and feasibility studies in the top dental specialty journals is suboptimal as many studies are not conducted with the intent of assessing feasibility, nor do they state progression criteria, and few report intent to proceed to a future trial.
- Misconceptions about PAFS can lead to them being poorly conducted and reported, which has economic and ethical implications. Thus, research ethics boards, funding agencies, and journals need to raise their standards for the conduct and reporting of PAFS, and resources should be developed to address misconceptions and help guide researchers on the best practices for their conduct and reporting.

## Introduction

Feasibility studies are defined as preliminary investigations conducted with the objective of assessing whether a future, larger, and more definitive study is feasible [1–3]. Pilot trials are a subset of feasibility studies, in which the future trial is implemented or partially implemented on a smaller scale to assess feasibility and inform its design and conduct [1–3]. However, the terms pilot trial and feasibility trial are often used synonymously in scientific literature and research guidelines [4, 5], so we will also use the terms indiscriminately in our investigation.

Pilot and feasibility studies (PAFS) are deemed almost a prerequisite to conducting a large trial, as their usage can improve trial design and be greatly effective at reducing resource waste [4, 6]. It is estimated that 85% of health research funds, equating to tens of billions of dollars, are wasted each year due to poor question selection, study design, reporting, and bias within reporting [7]. The United Kingdom’s National Institute for Health Research’s Research for Patient Benefit programme found that by employing 89 PAFS they saved about 20 million pounds that would have otherwise gone toward funding trials that were unfeasible [6]. It is evident that PAFS play an important role in reducing resource waste, but this is only true when their objectives aim to assess feasibility and when decisions to proceed to larger trials are made appropriately. Poorly designed PAFS present an ethical concern, as they could lead to misinformed conclusions about feasibility and thus lead researchers to proceed to conduct trials that are unfeasible. Participants of these potentially unfeasible trials often put their health at risk to volunteer in these studies and especially since many studies are publicly funded, researchers have a moral obligation to conduct high-quality research that is likely to be feasible.

The primary aim of a PAFS should be to assess the feasibility of a future definitive trial and their objectives can be broadly grouped into four domains: process, resources, management, and scientific [8]. Process-related objectives assess whether the steps that need to be taken to successfully conduct the main study are feasible, such as recruitment, retention, adherence, and data collection [3, 8]. Resource-related objectives seek to assess resource issues including time, money, and capacity [3, 8]. Management-related objectives seek to assess human and data management issues, for example challenges related to data variability, storage, and transfer [3, 8]. Scientific objectives seek to assess issues related to the intervention such as treatment safety, dose, and response [8]. Pilot trial findings inform the decision whether to proceed to a larger trial. This decision, as recommended by the Consolidated Standards of Reporting Trials (CONSORT) statement extension to pilot and feasibility trials and Thabane et al., should be made based on pre-specified progression criteria [8, 9]. It is important that these criteria are developed prior to the start of the trial to prevent introducing bias when progression criteria are added once the findings of the study are known. However, recent reports on PAFS found that only between 19.8% and 33.7% of pilot trial protocols reported progression criteria [3, 10].

Currently, it is unclear if PAFS in dentistry aim to assess feasibility and base their progression on pre-specified criteria. After conducting an informal review of the literature for studies assessing the design and reporting of PAFS in dentistry, we conclude that this issue has not been empirically investigated in the scientific literature. Thus, we sought to address this paucity of data by sampling PAFS in dental speciality journals and collecting information on their methods and reporting. The primary objective was to assess whether pilot trials, published after 2016, in the top dental speciality journals aim to assess feasibility and state clear progression criteria. The secondary objective was to conduct exploratory analysis to determine characteristics associated with studies that assess feasibility and state progression criteria.

## Methods

This review is reported in accordance with the Preferred Reporting Items for Systematic Reviews and Meta-Analyses (PRISMA) guidelines [11]. Since only secondary publicly available data is included ethics approval was not required.

### Search strategy and eligibility

We surveyed program directors from the ten recognized dental specialties in Canada, at the University of Toronto or the University of Western Ontario, and asked them to identify, based on their opinion, the top 3 journals in their specialty discipline. We identified 30 journals in total. We worked with a librarian from the University of Toronto dentistry library to develop a search strategy to identify and review PAFS published in the identified journals. We searched MEDLINE and EMBASE from 2017 to 2020, inclusive, using Ovid. These dates were selected since the CONSORT extension to PAFS was published in late 2016, which gave researchers publishing during this time an opportunity to implement the reporting guidelines described therein. For the full search strategy and journals that were searched from each specialty discipline see Additional file 1: Table S1.

A study was considered eligible if it stated it was a pilot or feasibility trial within the title or abstract, was conducted in humans, and published in English. Pilot or feasibility trial protocols, cadaveric studies, and conference abstracts were excluded. Abstract and full-text screening was conducted in duplicate by MIUK, HKB, CYS, RH, HAE, and KM, independently and with disagreements resolved by consensus.

### Data collection

Data was collected using a standardized data abstraction tool, developed based on the CONSORT extension to PAFS, by a single reviewer. To address our primary objective, we collected data on whether the studies stated some indication of intent to assess feasibility and their progression criteria.

To address our secondary objectives, we collected data on the methodological and general study characteristics, including the first author’s last name; study location; journal and year of publication; terms used in the title; randomization; trial design; number of study arms and sites; the type of data collected; if ethics approval was received; the type of intervention; funding sources; and reported sample size and rationale for the sample size. Our data collection tool also looked at the specific feasibility objectives, outcomes, and their categories (process, resource, management, or scientific); the specific progression criteria and their rationale; and whether the criteria were met. Finally, we collected information on whether the reports stated the need for a future trial, intent to proceed to a large trial, any proposed amendments to the study, whether hypothesis testing was reported and whether authors cautioned readers on the generalizability of their results.

### Data analysis

Data addressing the primary objective was used to calculate the proportion of studies in the sample with key reporting characteristics, including the terms used in the title, whether a future trial was needed, a future trial was to be pursued, amendments to the trial were described, and hypothesis testing was reported, with 95% confidence intervals (CIs). Descriptive statistics were used to summarize demographic and methodological characteristics of the studies. An exploratory analysis using multivariable binary logistic regression was used to determine characteristics associated with studies that report intent to assess feasibility, which was the dependent variable, and the independent variables being the study characteristics, including the terms used in the title of the study, year of publication, whether the trial was randomized, amendments to the trial design or implementation were proposed, and if hypothesis testing was done. Multivariable binary logistic regression results are reported as odds ratios (OR) with 95% CIs used to assess statistical significance. Analysis was performed using IBM SPSS Statistics version 26.

## Results

### Overview of studies

**Fig. 1:**
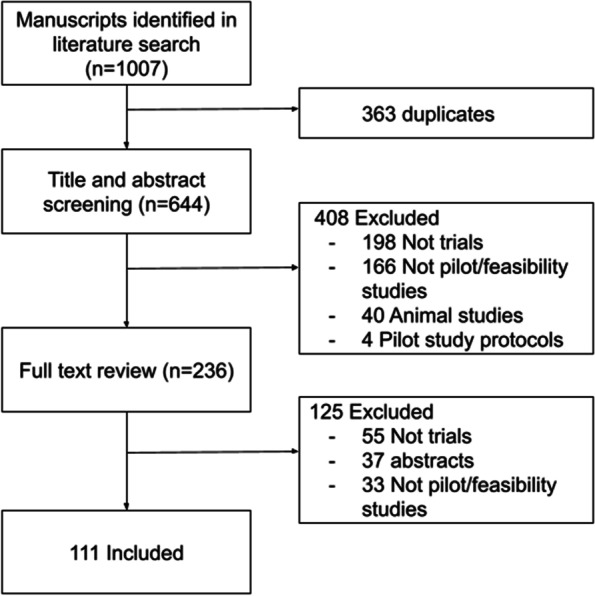
Flow diagram of the study inclusion process

An initial search of MEDLINE and EMBASE identified 1007 manuscripts as potential PAFS. After removing duplicates, using EndNote, and performing both abstract and full text screenings, 111 studies were deemed eligible and included (Fig. 1). Of the studies that were included, most of them were quantitative, non-industry funded, tested a surgical or procedural intervention and about half were randomized and used a parallel group design. The median sample size of the trials was 25, with a minimum sample size of three and a maximum of 392. Less than a quarter of reports provided a rationale for their sample size (reported use of a convenience sample was considered a rationale). See Table 1 for detailed descriptions of study demographics and Table 2 for descriptions of methodological study characteristics.

**Table 1:** General characteristics of studies included (*n* = 111)

Study characteristic	*n*, (%)
Trial location:	
Europe	49, (44.1)
Asia	28, (25.2)
North America	27, (24.3)
South America	4, (3.6)
Australia	3, (2.7)
Specialty discipline:	
Oral and maxillofacial surgery	26, (23.4)
Prosthodontics	19, (17.1)
Oral pathology and oral medicine	17, (15.3)
Periodontology	16, (14.4)
Pediatric dentistry	8, (7.2)
Orthodontics	7, (6.3)
Oral and maxillofacial radiology	6, (5.4)
Dental anesthesia	5, (4.5)
Dental public health	5, (4.5)
Endodontics	2, (1.8)
Year of publication:	
2017	33, (29.7)
2018	24, (21.6)
2019	22, (19.8)
2020	32, (28.8)

**Table 2:** Methodological characteristics of studies included (*n* = 111)

Study characteristic	*n*, (%)
Randomized	54, (48.6)
Quantitative	103, (92.8)
Qualitative	0, (0)
Mixed	8, (7.2)
Trial design:	
Parallel group	57, (51.4)
Single arm	41, (36.9)
Split mouth	8, (7.2)
Cross over	3, (2.7)
Cluster	2, (1.8)
Intervention type:	
Surgery/procedure	65, (58.6)
Drug	26, (23.4)
Material	4, (9.0)
Counseling/lifestyle	9, (8.1)
Industry funded	10, (9.0)
Stated rationale for sample size	25, (22.5)
Median sample size (min, max)	25, (3, 392)

### Assessment of feasibility and progression criteria

Half the studies, 51.4% (95% CI 41.7–61.0%), stated intent to assess feasibility, of which almost all of them, 49.5% (95% CI 39.9–59.2%) of total studies, stated specific feasibility objectives. The most common feasibility objective was scientifically related with 45.0% of studies having investigated these. This was followed by process, resource, and management related objectives with 17.1%, 6.3%, and 0.9% of studies having investigated these, respectively. Note that a study assessing more than one type of feasibility objective was counted multiple times, once in each category. Of the trials assessed, 45.9% had feasibility outcomes, with 38.7% of studies having analyzed these outcomes quantitatively, no studies used strictly qualitative assessment, and 7.2% used both quantitative and qualitative analysis. No studies reported progression criteria to help guide the decision of whether to proceed to a larger trial.

### Reporting characteristics and their association with assessment of feasibility

Most trials, 74.8% (95% CI 65.6–82.5%), used the terms “pilot” or “feasibility” in their titles; 82.9% (95% CI 74.6–89.4%) of studies stated there is a need for a future trial but only 9.0% (95% CI 4.4–15.9%) of total studies stated intent to pursue funding for or proceed to a larger trial. About half the reports, 52.3% (95% CI 42.6–61.8%), described amendments to the design or implementation of the trial to improve feasibility or rigor of future studies and 53.2% (95% CI 43.4–62.7%) of studies reported hypothesis testing without cautioning readers on the generalizability of the results. See Table 3 for further details on reporting characteristics.

**Table 3:** Proportion of studies with key reporting characteristics (*n* = 111)

Reporting characteristic	Percent of studies (95% confidence interval)
Used the terms “pilot” or “feasibility” in their titles	74.8% (65.6–82.5%)
Stated there is a need for a future trial	82.9% (74.6–89.4%)
Stated intent to pursue funding for or proceed to a larger trial	9.0% (4.4–15.9%)
Stated potential amendments to the design or implementation of the trial	52.3% (42.6–61.8%)
Did not report any hypothesis testing	2.25% (1.51–3.14%)
Reported hypothesis testing while cautioning readers on the generalizability of the results	24.3% (16.7–33.4%)
Reported hypothesis testing without cautioning readers on the generalizability of the results	53.2% (43.4–62.7%)

Results of the exploratory logistic regression modeled the odds of whether studies had feasibility objectives based on the terms used in their titles, year of publication, randomization, whether amendments were proposed, and reporting of hypothesis testing. We found that studies that used the terms “pilot” or “feasibility” in their title were less likely to assess feasibility, with an OR of 0.310 (95% CI 0.103–0.930; *p* = 0.037). We also found that trials reporting amendments to their design or implementation were more likely to assess feasibility, with an OR of 3.669 (95% CI 1.268–10.615; *p* = 0.016). Trials that conducted hypothesis testing were significantly less likely to assess feasibility, and among them, trials that cautioned readers on the generalizability of their results had an OR of 0.038 (95% CI 0.005–0.264; *p* < 0.001); and trials that did not caution readers on the generalizability of their results had an OR of 0.043 (95% CI 0.008–0.238; *p* = 0.001). The OR of all independent variables in the model, with their respective confidence intervals, can be found in Table 4.

**Table 4:** Binary logistic regression model presenting the odds of assessing feasibility (*n* = 111)

Estimated category	Reference category (odds ratio = 1)	Odds ratio (95% confidence interval)
Used the terms “pilot” or “feasibility” in the title	Did not mention “pilot” or “feasibility” in the title	0.310 (0.103–0.930)
Published in 2018	Published in 2017	1.156 (0.304–4.399)
Published in 2019	Published in 2017	0.801 (0.212–3.028)
Published in 2020	Published in 2017	1.630 (0.482–5.512)
Randomized trials	Non-randomized trials	0.530 (0.198–1.415)
Proposed amendments to improve the trial	Did not propose amendments to improve the trial	3.669 (1.268–10.615)
Conducted hypothesis testing without cautioning readers on generalizability of results	Did not conducted hypothesis testing	0.043 (0.008–0.238)
Conducted hypothesis testing and cautioned readers on generalizability of results	Did not conducted hypothesis testing	0.038 (0.005–0.264)

## Discussion

This is the first study, to our knowledge, assessing the reporting and methods of PAFS in dentistry based on the CONSORT extension [9]. We found that many studies failed to include important items from the CONSORT statement, suggesting there are meaningful gaps in the reporting and design of PAFS in the dental literature. This is perhaps due to a lack of awareness on the intended purpose and methodologies of PAFS. Considering that the field of pilot and feasibility studies is still in its early stages, challenges can be expected but they should serve to raise awareness of the issues and promote the development of guidelines and resources to improve the conduct and reporting of PAFS.

### Pilot trials and clinical decision-making

According to the CONSORT extension, it is important for PAFS to identify as such in their titles to allow them to be easily identified from search criteria and indexed in electronic databases [9]. It is also important because it helps readers identify the studies as being pilots and thereby informs them that any efficacy data presented should be interpreted with caution, as it is likely not generalizable to inform clinical decision-making. We found only 74.8% of the studies examined used the terms “pilot” or “feasibility” in their titles. This is less than other reports, as a review of cluster randomized controlled pilot trials and a review of pilot studies in clinical rehabilitation found 83% and 87% of studies used the terms “pilot” or “feasibility” in their titles, respectively [12, 13]. Although, it appears pilot trials in specialty dentistry journals are not far behind other disciplines, this is concerning since it could lead to some PAFS being misidentified and not included in reviews of the literature on pilot studies. It can also lead readers to believe the PAFS are assessing efficacy when they are often not powered to do so, as we found the median sample size to be 25. Thus, it is important for researchers and journals to ensure the titles of PAFS are labeled appropriately to enable ease of identification and thereby avoid clinical decision-making based on underpowered data.

There is also concern that PAFS generally place too much emphasis on hypothesis testing, despite being underpowered to reach generalizable conclusions on efficacy [14–16]. When PAFS conduct hypothesis testing, the results should be interpreted with caution, as with small sample sizes there is likely to be imbalance in pre-randomization covariates and confidence intervals are likely to be imprecise [15]. When formal power calculations have not been performed, the results of these studies are susceptible to the imbalances and imprecisions previously mentioned [15], which is particularly relevant since we found only 22.5% of studies provided a rationale, sample size calculation, or stated the use of a convenience sample for their sample sizes. Concerns of hypothesis testing in PAFS are further evidenced by our results, as over half (53.2%) the PAFS assessed conducted hypothesis testing without cautioning readers on the generalizability of the results. These reporting practices are incongruent with the CONSORT extension [9], which states generalizability of the findings ought to be reported. These incongruencies raise questions over the potential for inappropriate use of underpowered pilot data to make clinical decisions. Furthermore, these concerns are exacerbated at this point in time, in which authors argue we live in a “post-truth society” where scientists regularly encounter targeted media and social media campaigns of fake news, misinformation, and disinformation [17].

### Assessment of feasibility and misconceptions about pilot trials

Another issue of concern is that only 51.4% of the studies stated intent to assess feasibility, when this should be, by definition, the intended purpose of all PAFS and the distinction between them and other trials. This is also not in accordance with the CONSORT extension [9], which states that feasibility outcomes are to be pre-specified and well defined. Moreover, these results are less than other reports, as a review of pilot studies approved by a single research ethics board, pilot studies in clinical rehabilitation, and pilot trials in physical activity journals found 73.9%, 58%, and 46% of studies performed some assessment of feasibility, respectively [3, 13, 18]. A review of cluster randomized controlled pilot trials also found 66% of studies had feasibility as their primary objective [12]. This suggests that pilot studies are being misused or mislabelled, perhaps due to lack of understanding, from researchers and journals, of their intended purpose. This theory might explain the results of our logistic regression, which found studies that used the terms “pilot” or “feasibility” in their title were 69% less likely to assess feasibility, with an OR of 0.310 (95% CI 0.103–0.930; *p* = 0.037).

However, why does it appear that reporting of PAFS in dentistry is lacking, compared to other disciplines? Historically, oral medicine has had poor overall quality of published studies, which has lead to challenges in developing evidence based clinical recommendations [19]. Baccaglini et al. suggest that dentists and dental researchers may not have been as exposed to guidelines for reporting trials, due to lack of methodological articles in journals most commonly read by dentists; and that guidelines for reporting trials may be misunderstood as applying only to the publication of trials and not the planning phases. Trying to implement guidelines for reporting PAFS after the data is collected can be problematic because the outcomes measured are unlikely to be related to feasibility, and thus to retroactively change the objectives to assess feasibility after the study has been completed may not be practicable. Thus, the same principles described by Baccaglini et al. could explain why the reporting of PAFS in dentistry appears to be lacking compared to other disciplines.

In the present study, we found what appeared to be blatant misconceptions of the term pilot study. There was a trend that some studies with a low sample size would claim to be pilot studies, citing the low sample size as the reason. This might have been done to excuse the criticism of being underpowered or because of misconceptions on the purpose of PAFS. One study claimed, “one of the weaknesses of this study was that we were not able to accrue the pre-specified number of patients, which reduced the power of the study. Therefore, it is considered a pilot study” [20]. Based on this article and the general trend, it appears that researchers are misinformed of the definition and purpose of pilot studies, which is also supported by other reports [8, 16]. Thus, resources should be developed to help address any misconceptions and researchers should be made aware that small studies also have a role in the literature, for instance, they can be valuable for hypothesis testing and challenging common beliefs and practices [21].

### Progression criteria

Pre-defined progression criteria are important for unbiased evaluation of whether a PAFS should proceed to a larger study, proceed with modifications, or not proceed due to serious concerns of feasibility issues. We found that none of the studies assessed reported any progression criteria, which pales in comparison to findings from a review of randomized PAFS protocols which found 19.8% of studies reported progression criteria [10]. This raises the question: how do researchers conducting PAFS in dentistry determine whether to proceed to a future trial? Our results suggest that researchers, journals, and research ethics boards are potentially unaware of the importance of progression criteria or how they can be used. Thus, there is likely heterogeneity in how researchers decide whether to proceed to a future trial, which can lead to conduct of trials that are unfeasible or fail to proceed to trials that would have been feasible—both of which result in wasted resources.

One potential reason that many PAFS do not report progression criteria is because the trials are not intended to assess feasibility to begin with. We found that about half (51.4%) of the trials examined stated intent to assess feasibility and thus we would reasonably assume that only this half could have stated progression criteria. As such, perhaps the lack of progression criteria stems from misinformation on the purpose of PAFS or poorly selected objectives or study design. This again highlights the need for resources and training for researchers, journals (editors and peer reviewers), and research ethics boards on the conduct of PAFS.

Interestingly, 82.9% of studies stated the need for a future trial and yet only 9.0% stated intent to proceed to a future trial or pursue funding for one. Why are authors not reporting intent to proceed to a future trial when they claim it is needed? It could be that the studies are not feasible or perhaps they did not assess feasibility to begin with, as we found only about half of them did. It could also be because there are no progression criteria to help guide the decision to pursue a future study, so authors find it difficult to make this decision and thereby do not report it. The use of progression criteria could provide clarity in making unbiased assessments of whether to proceed to a larger study, or identify aspects of the trial that can be modified to enhance feasibility, and lead to more authors reporting their intentions for whether to proceed to a future trial.

### Limitations, areas of future research, and resources

There were various limitations of the present study. We searched pre-specified journals which introduced a selection bias and thereby limits the generalizability of our findings to PAFS in dental speciality journals and dentistry as a whole. The journals searched were based on the opinions of program directors, which was done to limit the number of articles reviewed, due to resource constraints. This selection bias could skew the results to suggest better or worse reporting of PAFS. If the more prestigious journals included in our search adhere more strictly to the CONSORT extension than other journals, then our results might suggest better reporting than the overall landscape of PAFS in dental specialty journals. However, if misconceptions, like the need for hypothesis testing are widespread, then perhaps well reported PAFS would not be accepted to the journals searched in this study, which would skew our results to suggest worse reporting than PAFS published in other, lower impact, journals. Another limitation of this study was that the final data extraction was conducted by a single reviewer (MIUK), due to time and resource constraints. Furthermore, our analysis was limited by the nature of our data, as after considering the assumptions of logistic regression and removing variables with multicollinearity, we were only able to include five independent variables in the model. Moreover, we were only able to develop a regression model for one of our two primary outcomes (whether the trial intended to assess feasibility), as there were no observations of studies reporting progression criteria. Nonetheless, this study is the first look at the quality of reporting of PAFS in dentistry and highlights important issues that are consistent with other findings in the literature on pilot studies.

Future research should focus on the reporting of PAFS in dentistry as a whole and developing resources for researchers, journals, and research ethics boards to address misconceptions on pilot studies and assist in the planning and reporting of pilot trials based on best practices. A recent review found that CONSORT statement endorsement by journals was associated with more complete reporting of randomized clinical trials in dentistry [22]. Future work could assess whether this holds true with respect to the reporting of PAFS in dentistry as well. As the field of pilot and feasibility studies evolves, it is important to identify issues and areas of concern as well as their etiology.

To date, there are some resources available to help design, evaluate and report PAFS. The CONSORT extension [9] along with other editorial pieces can serve as a guide for researchers [8, 23]. The Queen Mary University of London also has a website dedicated to support researchers conducting PAFS and related methodological research [24]. Many PAFS do not get published or have been difficult to publish [25]; however, researchers have an ethical and scientific obligation to attempt to publish the results of their pilot studies [8]. The journal, *Pilot and Feasibility Studies,* aims to provide researchers a dedicated place to report their PAFS and related methodological research. Although these resources are in place and demonstrate a major step in the right direction, it will take time for researchers, journals, and research ethics boards to implement these recommendations and be made aware of the best practices for designing, implementing and reporting PAFS.

## Conclusion

Based on our limited assessment of PAFS in 30 dental specialty journals, it appears there are various concerns and issues with respect to the reporting and conduct of pilot studies in dentistry. Many pilot trials in dentistry are not conducted with the intent of assessing feasibility, while the significance of their results are reported without cautioning readers on their generalizability. No progression criteria were found, which accompanied few reports of intent to proceed to a future trial. There appears to be many misconceptions with respect to PAFS in dentistry, which can lead to poor design, conduct and reporting of pilot trials, which has meaningful economic and ethical implications. Research ethics boards, funding agencies, and journals need to raise their standards for the conduct and reporting of PAFS and resources should be developed to address misconceptions and help guide researchers on the best practices for conduct and reporting of pilot studies.

## Supplementary Information

**Additional file 1: Table S1.** Search strategy for EMBASE and MEDLINE. **Table S2.** The journals searched for dental speciality.

## Data Availability

The datasets used in this study are available from the corresponding author upon request. They encourage collaborations.
