# Supplementary material for: The reporting of pilot and feasibility studies in the top dental specialty journals is suboptimal
Source: Pilot Feasibility Stud. 2022 Oct 4;8:224. doi: 10.1186/s40814-022-01182-1 (PMC9531373; doi:10.1186/s40814-022-01182-1)
Supplement: Supplementary file 1 — Additional file 1: Table S1. Search strategy for EMBASE and MEDLINE. Table S2. The journals searched for dental speciality. [file 40814_2022_1182_MOESM1_ESM.docx]

**Appendix 1**

Table 1: Search strategy for EMBASE and MEDLINE.

| **No** | **Search terms** |
| --- | --- |
| 1 | "Oral Surgery Oral Medicine Oral Pathology and Oral Radiology".jn. OR “Oral Radiology”.jn. OR "Journal of Oral Medicine".jn. |
| 2 | “Oral Oncology".jn. OR “Oral Diseases".jn. OR “Journal of Oral Pathology and Medicine".jn. |
| 3 | "Journal of Oral and Maxillofacial Surgery".jn. OR "International Journal of Oral and Maxillofacial Surgery".jn. OR "Journal of Cranio Maxillo Facial Surgery".jn. |
| 4 | “Journal of Endodontics".jn. OR “International Endodontic Journal".jn. OR “Dental Traumatology".jn. |
| 5 | “Journal of Clinical Periodontology".jn. OR “Journal of Periodontology".jn. OR “Journal of Periodontal Research".jn. |
| 6 | “American Journal of Orthodontics and Dentofacial Orthopedics".jn. OR “Angle Orthodontist".jn. OR “European Journal of Orthodontics".jn. |
| 7 | “Journal of Prosthetic Dentistry".jn. OR “International Journal of Prosthodontics".jn. OR “International Journal of Oral and Maxillofacial Implants” |
| 8 | “Pediatric Dentistry".jn. OR “Dental Traumatology".jn. OR “International Journal of Pediatric Dentistry".jn. |
| 9 | “Anesthesia Progress".jn. OR “Journal of Japanese Dental Society of Anesthesiology".jn. OR “Journal of Dental Anesthesia and Pain Medicine".jn. |
| 10 | “Journal of Dental Research".jn. OR “Journal of Public Health Dentistry".jn. OR “Community Dentistry and Oral Epidemiology".jn. |
| 11 | 1 OR 2 OR 3 OR 4 OR 5 OR 6 OR 7 OR 8 OR 9 OR 10 |
| 12 | (Pilot OR Feasibility).kw,tw. |
| 13 | Feasibility Studies/ OR Pilot Projects/ |
| 14 | 12 OR 13 |
| 15 | 11 AND 14 |
| 16 | Limit 15 to yr=”2017-2021” |

*When searching MEDLINE “and” is replaced with “&”

Table 2: The journals searched for dental speciality.

| Specialty | Journals Searched |
| --- | --- |
| Oral and Maxillofacial Radiology | -Oral Surgery Oral Medicine Oral Pathology and Oral Radiology  -Oral Radiology  -Journal of Oral Medicine |
| Oral Medicine and Oral Pathology | -Oral Oncology  -Oral Diseases  -Journal of Oral Pathology and Medicine |
| Oral and Maxillofacial Surgery | -Journal of Oral and Maxillofacial Surgery  -International Journal of Oral and Maxillofacial Surgery  -Journal of Cranio Maxillo Facial Surgery |
| Endodontics | -Journal of Endodontics  -International Endodontic Journal  -Dental Traumatology |
| Periodontics | -Journal of Clinical Periodontology  -Journal of Periodontology  -Journal of Periodontal Research |
| Orthodontics | -American Journal of Orthodontics and Dentofacial Orthopedics  -Angle Orthodontist  -European Journal of Orthodontics |
| Prosthodontics | -Journal of Prosthetic Dentistry  -International Journal of Prosthodontics  -International Journal of Oral and Maxillofacial Implants |
| Paediatric Dentistry | -Pediatric Dentistry  -Dental Traumatology  -International Journal of Pediatric Dentistry |
| Dental Anaesthesia | -Anesthesia Progress  -Journal of Japanese Dental Society of Anesthesiology  -Journal of Dental Anesthesia and Pain Medicine |
| Dental Public Health | -Journal of Dental Research  -Journal of Public Health Dentistry  -Community Dentistry and Oral Epidemiology |
